# Supplementary material for: Pilot Study: Quantitative Photoacoustic Evaluation of Peripheral Vascular Dynamics Induced by Carfilzomib In Vivo
Source: Sensors (Basel). 2021 Jan 27;21(3):836. doi: 10.3390/s21030836 (PMC7865712; doi:10.3390/s21030836)
Supplement: Supplementary file 1 [file sensors-21-00836-s001.zip › SI/Supplementary Material-final.docx]

**Supplementary Materials**

Manuscript tile: Pilot Study: Quantitative Photoacoustic Evaluation of Peripheral Vascular Dynamics Induced by Carfilzomib *In Vivo*

**Authors:** Thi Thao Mai, Manh-Cuong Vo, Tan-Huy Chu, Jin Young Kim, Chulhong Kim, Je-Jung Lee, Sung-Hoon Jung*, and Changho Lee *

First, the small 9 ROIs were randomly selected in OR-PAM MAP images. All ROIs have same size [45 × 50] pixels. Second, the MAP image of each ROI was extract. Third, we implemented quantitative process to extract five parameters of vasculatures inside ROI.

1. ***In vivo* OR-PAM observation for the peripheral vasculatures after carfilzomib solution injection**


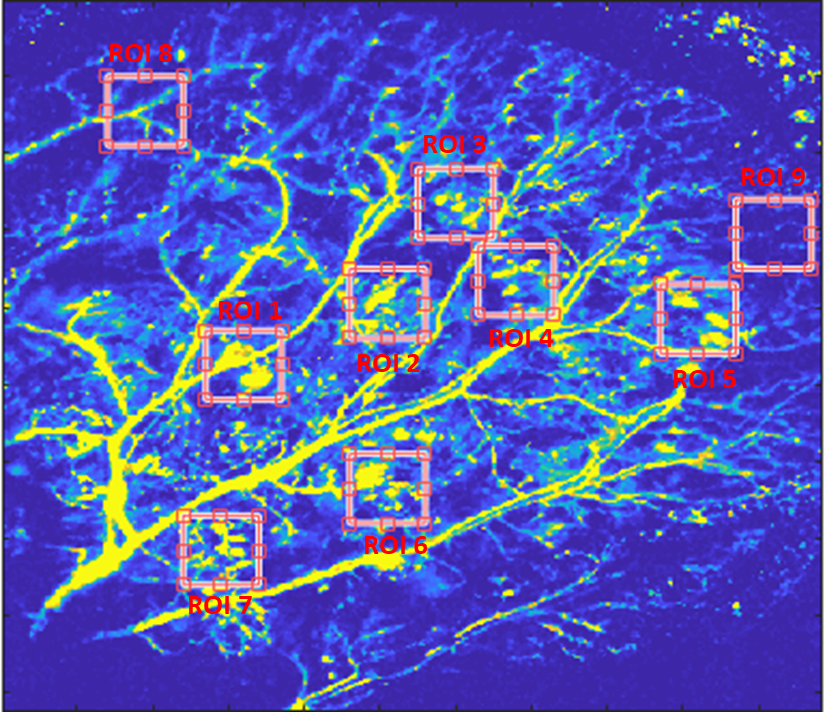


**Figure S1**. Selected small 9 ROIs after carfilzomib solution injection.


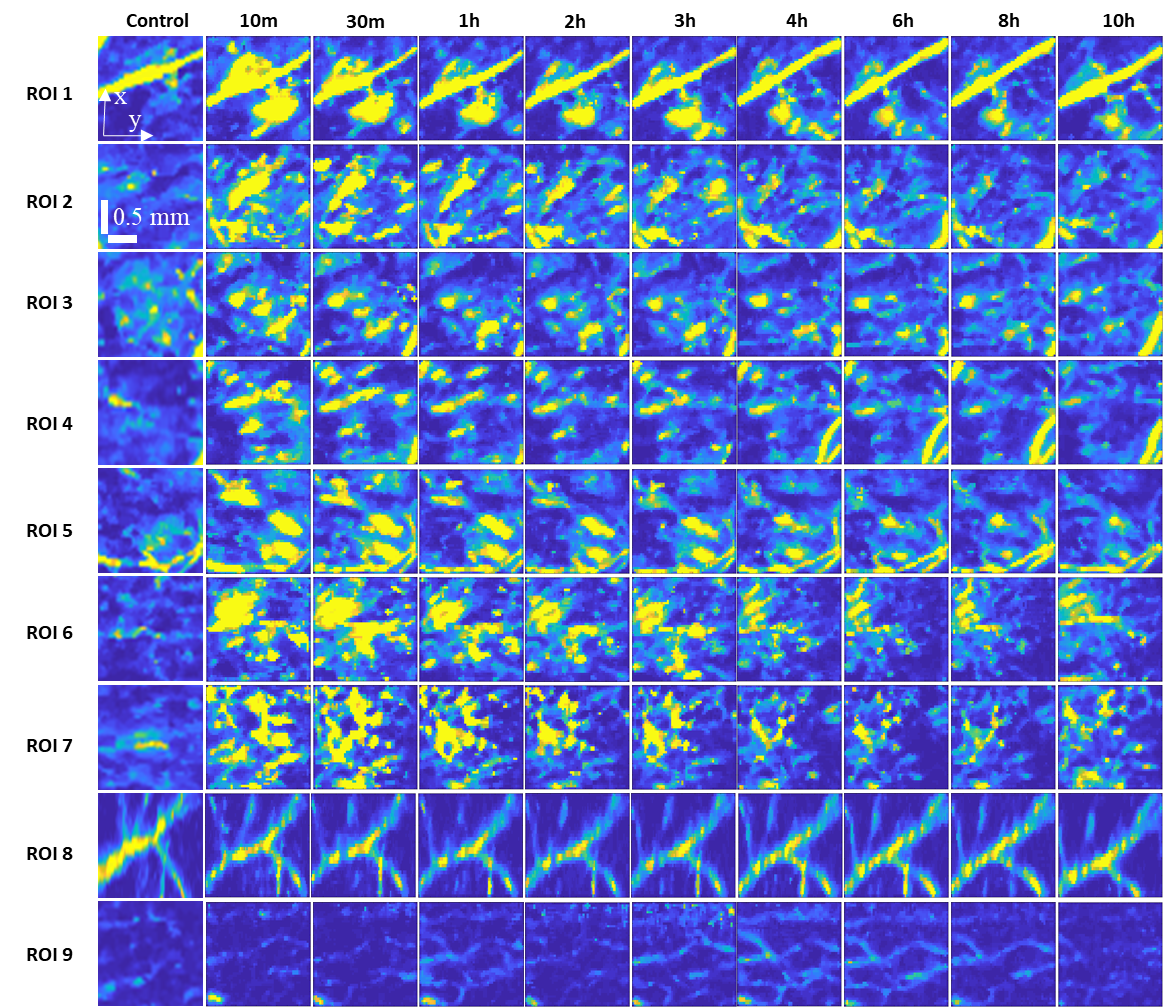


**Figure S2**. OR-PAM MAP images of 9 ROIs during 10 hours after carfilzomib solution injection.


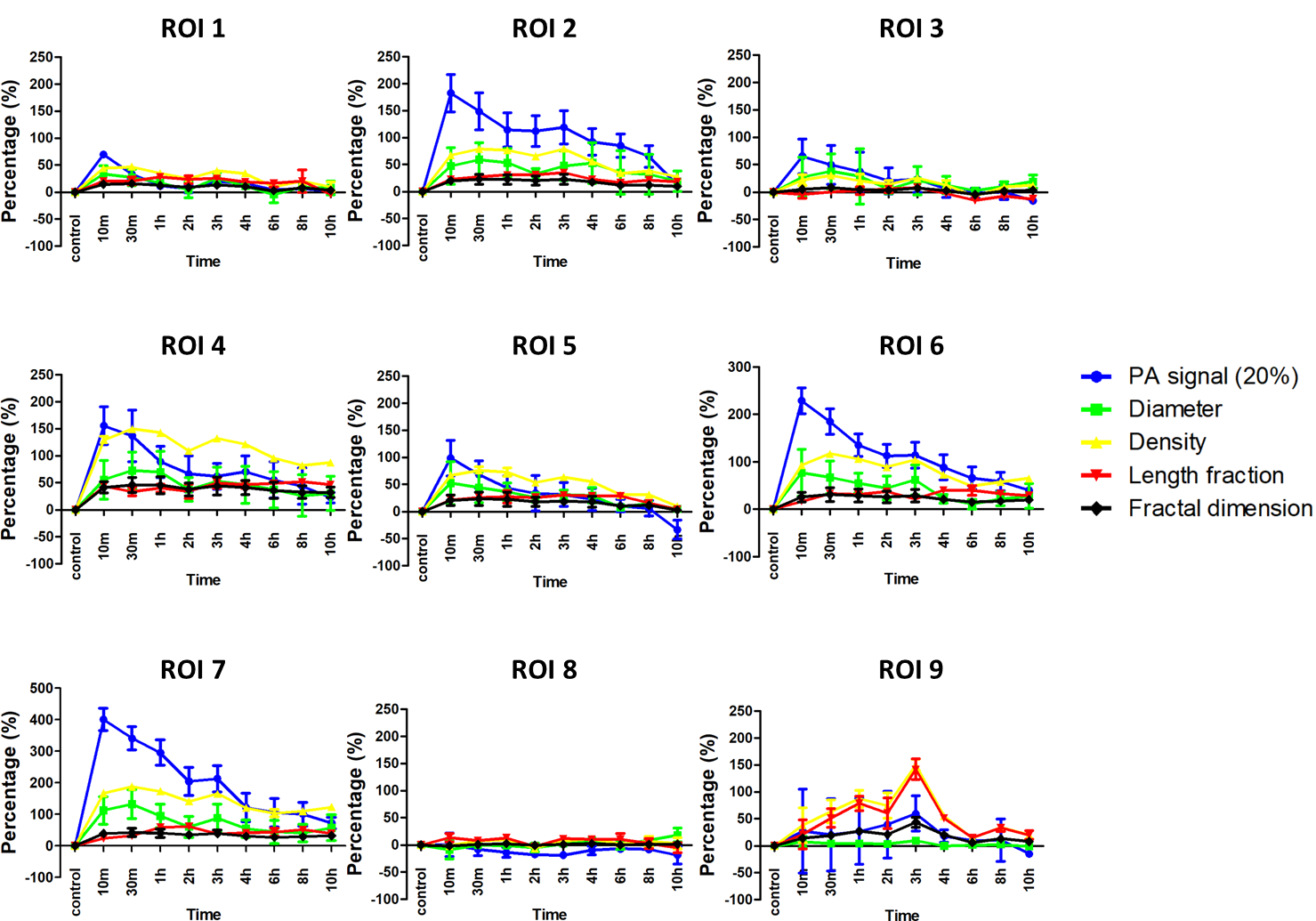


**Figure S3**. Quantitative evaluation of 9 ROIs within 10 hours after carfilzomib injection.

1. ***In vivo*** **OR-PAM observation for the peripheral vasculatures of after bortezomib solution injection**


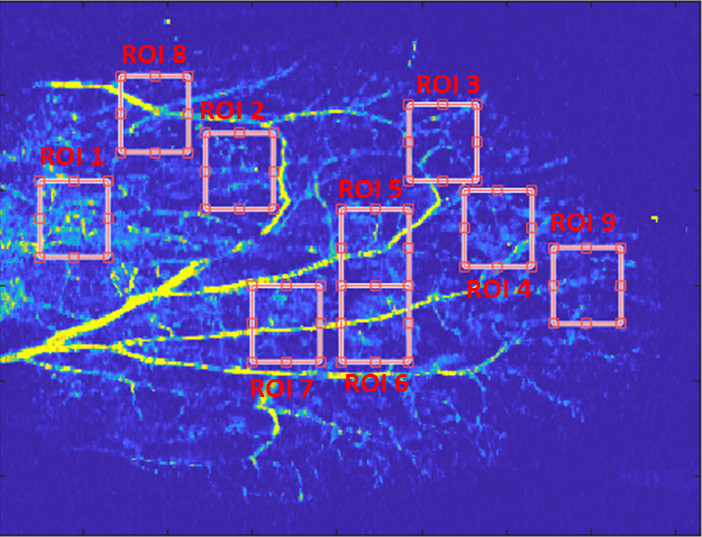


**Figure S4**. Selected small 9 ROIs after bortezomib solution injection.


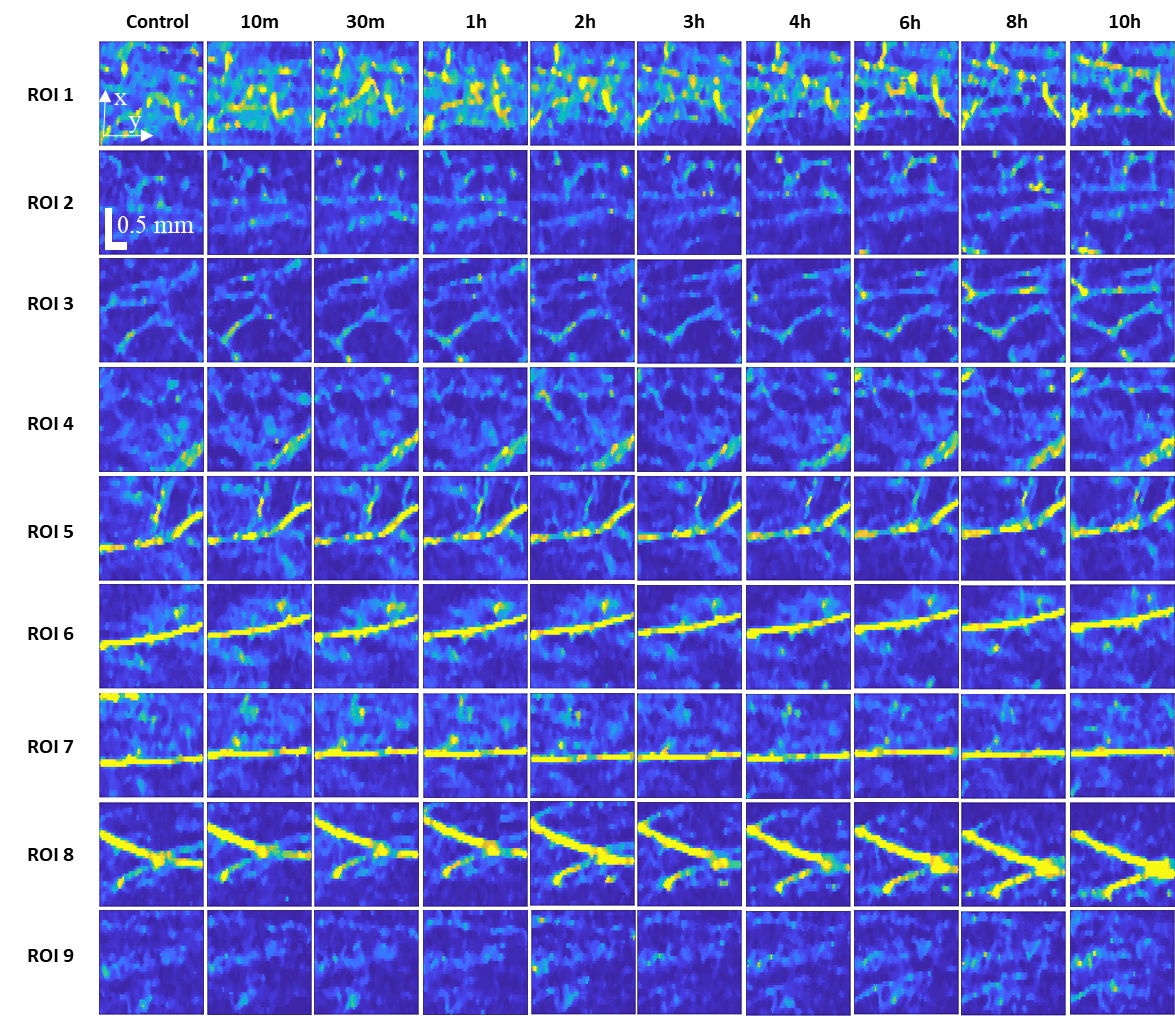


**Figure S5**. OR-PAM MAP images of 9 ROIs during 10 hours after bortezomib solution injection.


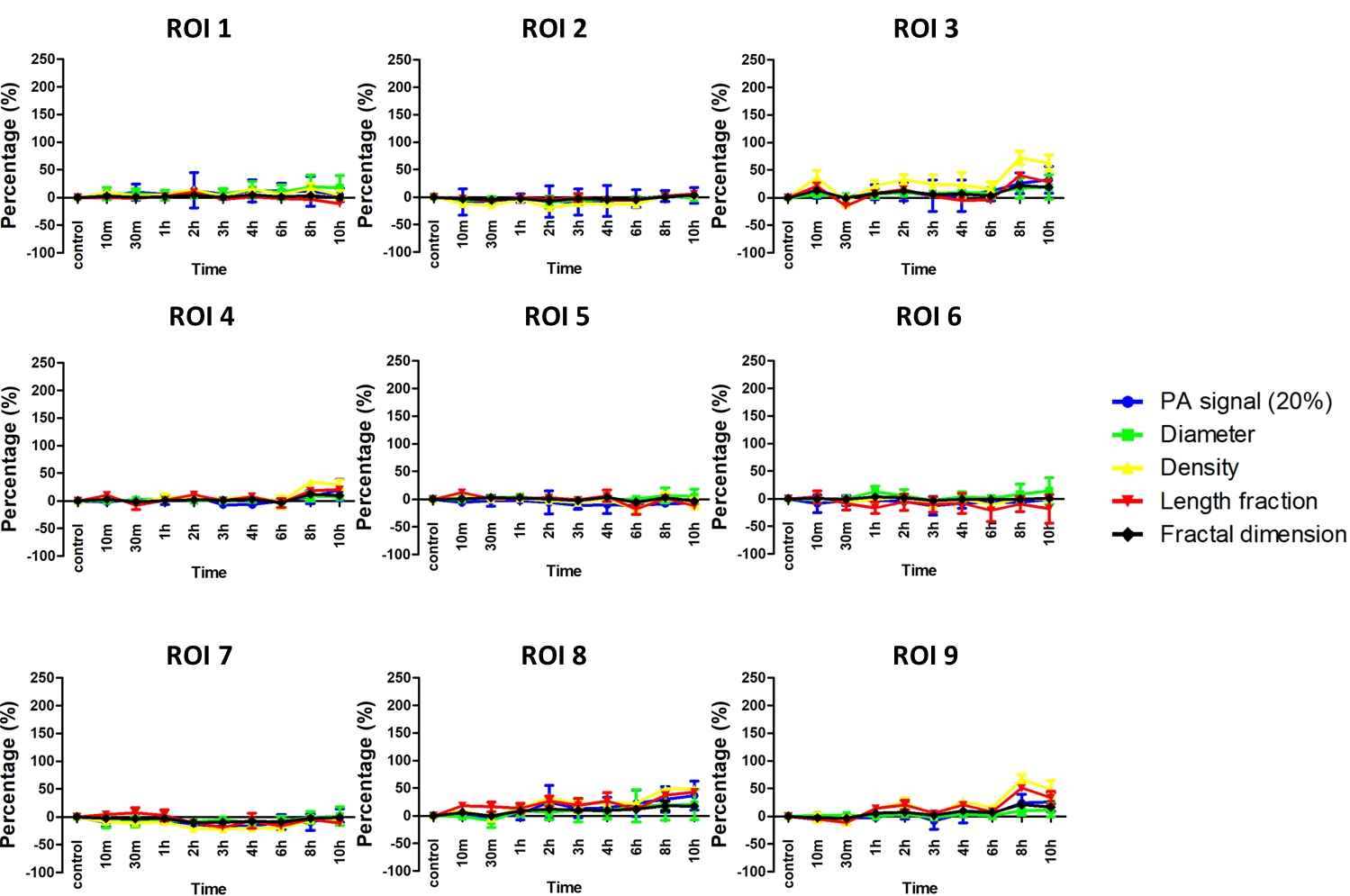


**Figure S6**. Quantitative evaluation of 9 ROIs within 10 hours after bortezomib solution injection.

1. ***In vivo* OR-PAM observation for the peripheral vasculatures of after PBS injection**


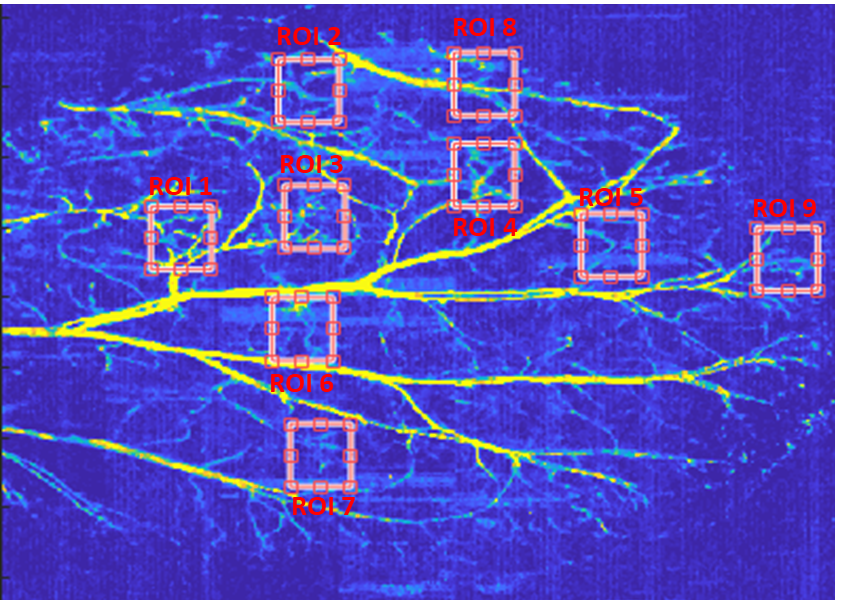


**Figure S7**. Selected small 9 ROIs after PBS injection.


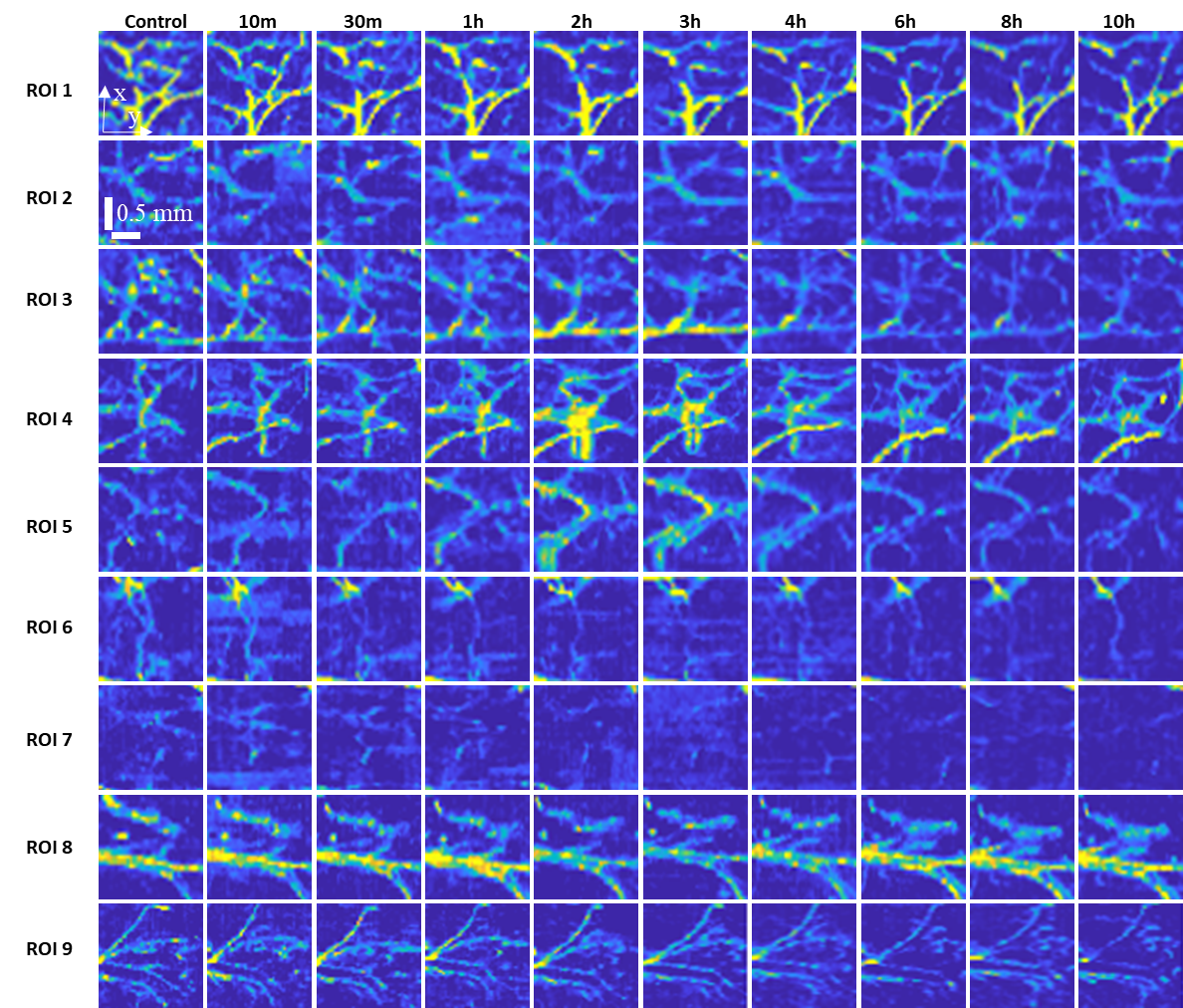


**Figure S8**. OR-PAM MAP images of 9 ROIs during 10 hours after PBS injection.


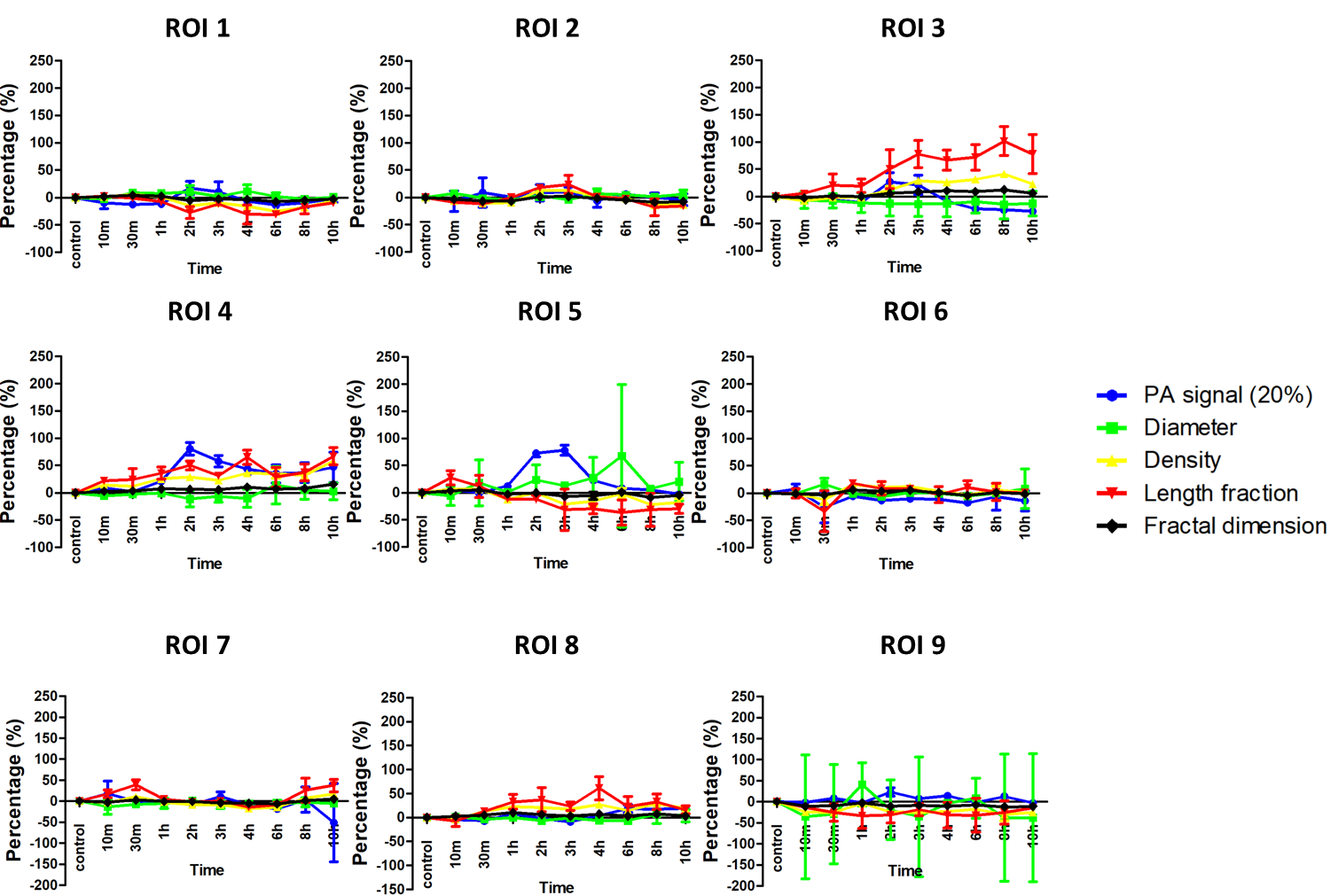


**Figure S9**. Quantitative evaluation of 9 ROIs within 10 hours after PBS injection.
